# CH_3_NH_3_PbI_3_ perovskite single crystals: surface photophysics and their interaction with the environment†
†Electronic supplementary information (ESI) available. See DOI: 10.1039/c5sc02542g


**DOI:** 10.1039/c5sc02542g

**Published:** 2015-09-17

**Authors:** G. Grancini, V. D'Innocenzo, E. R. Dohner, N. Martino, A. R. Srimath Kandada, E. Mosconi, F. De Angelis, H. I. Karunadasa, E. T. Hoke, A. Petrozza

**Affiliations:** a Center for Nano Science and Technology@Polimi , Istituto Italiano di Tecnologia , via Giovanni Pascoli 70/3 , 20133 , Milan , Italy . Email: giulia.grancini@epfl.ch ; Email: annamaria.petrozza@iit.it; b Dipartimento di Fisica , Politecnico di Milano , Piazza L. da Vinci, 32 , 20133 Milano , Italy; c Department of Chemistry , Stanford University , 337 Campus Drive , Stanford , California 94305 , USA; d Computational Laboratory for Hybrid/Organic Photovoltaics (CLHYO) , CNR-ISTM , Via Elce di Sotto 8 , I-06123 , Perugia , Italy; e Department of Materials Science and Engineering , Stanford University , 476 Lomita Mall , Stanford , California 94305 , USA

## Abstract

Here we identify structural inhomogeneity on a micrometer scale across the surface of a CH_3_NH_3_PbI_3_ perovskite single crystal. At the crystal edge a local distortion of the crystal lattice is responsible for a widening of the optical bandgap and faster photo-carrier recombination. These effects are inherently present at the edge of the crystal, and further enhanced upon water intercalation, as a preliminary step in the hydration of the perovskite material.

## Abstract 2

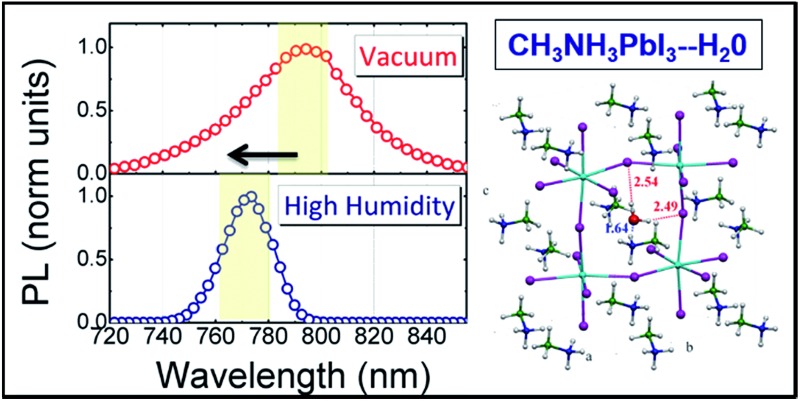
Structural inhomogeneity on a micrometer-scale across a CH_3_NH_3_PbI_3_ single crystal is responsible for a local modulation of the optical band gap, which is also highly sensitive to humidity.

## Introduction

Hybrid perovskite based solar cells have already exceeded a certified power conversion efficiency of 20.1%.^1,2^ However, a clear understanding of the relationship between the optoelectronic properties of these materials and their morphology is lacking due to the large degree of structural variability within samples. Hybrid perovskites are usually deposited as polycrystalline thin-films with variable mesoscale morphology.^3–5^ Depending on the growth conditions, the obtained grain size typically ranges from tens to thousands of nm.^3–5^ To overcome this issue, perovskite single crystals are the ideal sample for understanding the inherent properties of the material, providing insight into their full potential once embodied in optoelectronic devices. However, several recent studies have reported conflicting optical properties for MAPbI_3_ (MA = CH_3_NH_3_^+^) single crystals.^6–9^ Here we provide a unifying picture, by highlighting the variation of the structural and optical properties across the crystal surface at a micrometer scale and by elucidating its interaction with the environment. Indeed, the optical properties we reveal at the crystal edges more closely resemble those of polycrystalline MAPbI_3_ thin films used in optoelectronic devices.^10,11^ These local probes of the structure and photophysical dynamics of the single crystal edge sites provide a systematic method for studying and optimizing the active interfaces, which are of high technological impact in polycrystalline perovskite films.

## Results and discussion

**Fig. 1:**
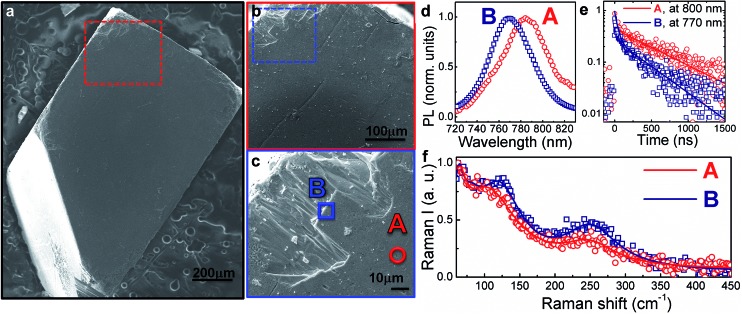
(a) Top view SEM image of a mm-sized MAPbI_3_ single crystal (see Fig. S2† for XRD data); (b) enlarged view of the SEM in the top-left area of (a); (c) higher-resolution image of the SEM in the top-left area of (b) where spot A (towards the centre of the crystal face) and B (at the edge of the crystal face) have been measured, respectively; (d) PL spectra on spot A and B upon excitation at 690 nm (fluence ∼ 10 μJ cm^–2^) under a N_2_ atmosphere; (e) PL time decays at 770 nm (peak of point B PL spectrum) and 800 nm (peak of point A PL spectrum), solid lines represent the fits (*y* = 0.5 exp(–*t*/*τ*_1_) + 0.2 exp(–*t*/*τ*_2_), *τ*_1_ = 630 ns, *τ*_2_ = 40 ns fits the decay at 800 nm; *y* = 0.2 exp(–*t*/*τ*_1_) + 0.44 exp(–*t*/*τ*_2_), *τ*_1_ = 500 ns, *τ*_2_ = 38 ns fits the decay at 770 nm). (f) Raman spectra from point B (blue squares) and point A (red dots) along with fitting results (solid lines). The measurement has been done under a N_2_ atmosphere.

Fig. 1a–c shows the SEM image of the single crystal and higher resolution images of the region investigated. Specifically, we probe points at the centre of the crystal face (point A) or at the crystal edge (point B). The local photoluminescence spectra and dynamics have been measured with a spatial resolution of ∼1 μm (micro-PL hereafter, see the Experimental section for details) with the aim to probe the optical properties of the single crystal surface at a local scale. Example PL spectra and decay dynamics from the centre of the crystal face and the edge (taken at points A and B, respectively) are presented in Fig. 1d and e. The PL spectrum blue-shifts and the PL decay accelerates when moving from the centre to the crystal edge. In particular, the higher-energy PL from the crystal edge (Fig. 1c, point B) consists mainly of a fast component (∼40 ns, see Fig. 1e), along with a minor contribution from a long-lived tail (∼600 ns). On the contrary, for the lower-energy PL from the crystal centre (Fig. 1c and e, point A) the long-lived component dominates over the fast decay. Though the actual shift cannot be accurately quantified due to re-absorption effects that cannot be fully disregarded, together, these observations suggest that the spatial variation of the PL spectra arises from energetic inhomogeneity across the crystal. Such a conclusion can be further corroborated by looking at the spatial variation of the transient reflectance (TR) signal across the crystal face, which probes the semiconductor band edge (by monitoring the band edge photobleaching upon carrier cooling – see Fig. S1 in the ESI† with relative discussion).

Considering that the excitation wavelength of 690 nm has an absorption depth of approximately 250 nm, surface effects (*e.g.*, dangling bonds, under-coordinated atoms) will dominate the micro-PL measurement, certainly significant when probing the surface edges. We suggest that these edge surface effects are responsible for the blue-shifted, faster decaying component of the PL. In polycrystalline perovskite thin films it has been demonstrated^11,12^ that deformation of the inorganic cage through interactions with the organic cations can compress the lattice, resulting in an increased bandgap.^11–15^ Specific vibrational modes have been assigned as evidence for these lattice structural distortions.^11,16,17^

To better understand the origin of the spatially inhomogeneous PL, we measured the micro-Raman spectra at a ∼1 μm scale resolution on exactly the same points at which the micro-PL spectra were measured (Fig. 1f; see the Experimental section for details on the set-up). Excitation at the centre (point A) and the edge (point B) of the crystal face leads to different spectra, suggesting a restructuring of the crystal surface at the edges. The former shows a peak at 109 cm^–1^, assigned to the Pb–I stretching mode^17^ and a broad band at 250 cm^–1^, previously assigned to the torsional mode of the MA cation.^11,17^ When moving to the edge, we observe that the frequency of the Pb–I stretching mode is blue-shifted (towards 120 cm^–1^) and the peak gains intensity, while the band at 250 cm^–1^ slightly shifts and gets more intense. A similar trend has been previously observed for MAPbI_3_ nanocrystals grown within a mesoporous oxide scaffold, which were reported to be more distorted with respect to micrometer-size MAPbI_3_ crystallites deposited on a flat glass substrate.^11^ This result suggests that lattice strain at the crystal edges can be responsible for the blue-shifted PL. The above observations indicate that the edges of the MAPbI_3_ single crystal differ optically, energetically and structurally from the centre of the crystal face. This is not surprising, considering that defects and lattice reconstructions are common at crystal edges. Note also that the micro-PL spectra we measure at the crystal edges, rather than the crystal centre PL, more closely resemble the usual PL spectra observed for MAPbI_3_ polycrystalline thin films.^10,11,19^ Thus, we believe that the distorted crystal surface provides a model for the properties of polycrystalline thin films with small grains where surface effects at high defect densities, grain boundaries and active interfaces dominate and dramatically impact the device operation.^18^

**Fig. 2:**
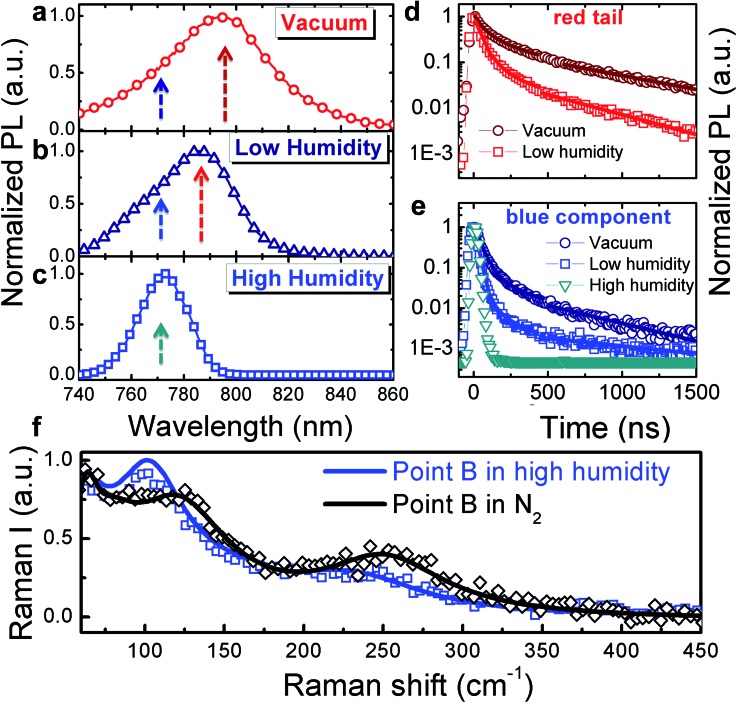
Normalized PL spectra (a–c) and dynamics (d and e, respectively) at the wavelengths indicated by the arrows in (a–c), collected under vacuum (a), under a low humidity atmosphere (b) and under high humidity (c); fluence ∼ 10 μJ cm^–2^. The solid lines in (2d and 2e) represent exponential fits: (*y* = 0.9 exp(–*t*/*τ*_1_) + 0.1 exp(–*t*/*τ*_2_)), *τ*_1_ = 35 ns *τ*_2_ = 240 ns fits the decay in vacuum; *y* = 0.95 exp(–*t*/*τ*_1_) + 0.05 exp(–*t*/*τ*_2_), *τ*_1_ = 26 ns, *τ*_2_ = 200 ns fits the decay under low humidity and *y* = exp(–*t*/*τ*), *τ* = 22 ns fits the decay under high humidity. (f) Comparison of the Raman spectra at point B, under dry N_2_ *versus* under a high humidity environment.

Because the surface is exposed to the surrounding atmosphere, we investigated how the optical properties of the single crystal surface are sensitive to environmental interaction, in particular to the presence of moisture. The interaction between MAPbI_3_ and water is of particular concern as moisture has been shown to dramatically impact not only the perovskite film growth and crystallization dynamics^20–25^ but also accelerate material degradation.^25–28^ Fig. 2 shows the macro-PL spectra measured on the whole crystal surface (a) under vacuum, (b) under low humidity (dry air with water content <10 ppm mol^–1^) and (c) under high humidity (ambient conditions, ∼50% relative humidity) (see Fig. S3† for a cartoon of the excitation–collection geometry).

Here we are probing the signal from the whole crystal surface, including the edges. As shown, when under vacuum (Fig. 2a), the PL spectrum appears broad and asymmetric. Upon increasing exposure to humidity, the blue component becomes dominant (Fig. 2b and c) resulting in a sharp spectrum (FWHM ∼ 22 nm) peaking at 770 nm and the PL decay dynamics are shortened (Fig. 2d and e) showing a complete quenching of the longer-wavelength emission under ambient humidity. The PL spectra were taken within a few minutes of changing the test chamber atmosphere, indicating that the spectral changes are rapid. Such behaviour indicates that under ambient conditions, the reconstruction of the crystal surface induced by lattice distortion becomes more significant.

We hypothesize that the strained surface region grows and extends deeper into the crystal upon moisture exposure. Note that this phenomenon is partially reversible as shown by the recovery of the PL decay dynamics after storing the sample back under vacuum (Fig. S4†). To validate our hypothesis that exposure to the environment leads to a local distortion at the crystal surface we measured the micro-Raman spectra at the edge of the crystal in the presence of ambient humidity (Fig. 2f). In particular, the peak at 109 cm^–1^ shifts to lower energy and increases in intensity giving evidence for local distortion.^11,17^ Concomitantly, the MA cation mode at 250 cm^-1^ red shifts as well. Overall, this behaviour can be the result of water molecule interactions through hydrogen bonding with the organic cations and the halide ions. This is particularly relevant at the crystal edge. The centre of the crystal face, indeed, is less affected by interaction with humidity (see Fig. S5 in the ESI†).

We remark here that the widening of the band gap observed upon moisture exposure along with structural distortion happening at the crystal edges are distinct from the optical and structural features related to perovskite conversion into its hydrated phases.^21,23,26^ Hydrated forms of MAPbI_3_, such as the (CH_3_NH_3_)_4_PbI_6_·2H_2_O compound consisting of isolated PbI_6_^4–^ octahedra,^21,23,26,29^ are indeed pale yellow in colour, and exhibit a bandgap of over 3 eV and a zero-dimensional structure.^21,23,26^ We therefore believe that what we observe is likely an initial step of structural reorganization towards the conversion to a hydrated phase.

**Fig. 3:**
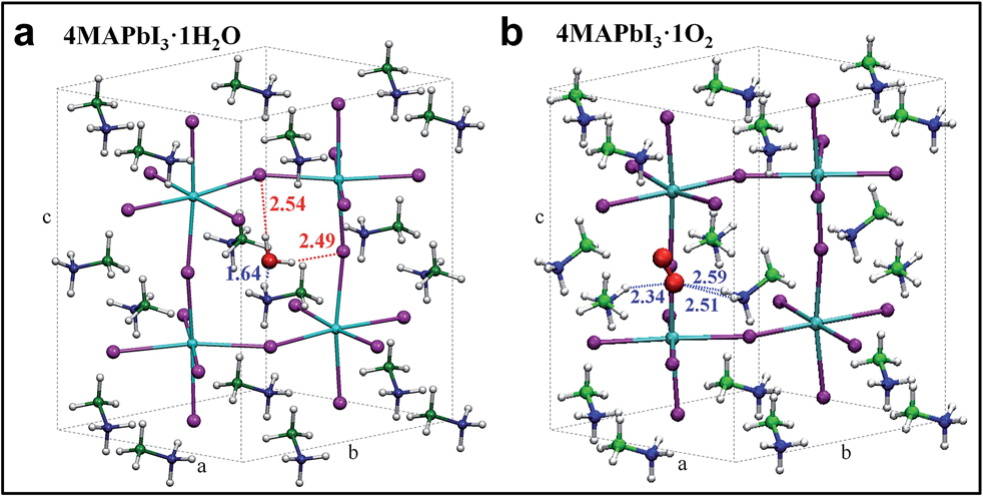
Optimized geometry structures for 4MAPbI_3_·1H_2_O (a) and 4MAPbI_3_·1O_2_ (b). Pb = light blue, I = violet, green = carbon, blue = nitrogen, red = oxygen, white = hydrogen.

As evidenced by *ab initio* molecular dynamics simulations,^30^ water molecules can permeate across the perovskite structure to form a partly hydrated phase. Based on this preliminary observation, we further computationally simulated the possible phases obtained from stepwise perovskite hydration. To investigate how water and oxygen molecules can interact with the hybrid perovskite we calculated the formation energies for 4MAPbI_3_·*n*H_2_O and 4MAPbI_3_·O_2_, (Fig. 3a and b, respectively), by scalar-relativistic Perdew–Burke–Ernzerhof (PBE) calculations employing van der Waals corrections. Starting from a tetragonal unit cell, consisting of four MAPbI_3_ units, we sequentially add either one oxygen or up to four water molecules. The calculated relaxed atomic positions for 4MAPbI_3_·H_2_O and 4MAPbI_3_·O_2_ are shown in Fig. 3. Both structures exhibit an increase in cell volume of ∼11–12 Å^3^ with respect to MAPbI_3_. While the incorporation of one water molecule preserves the original tetragonal cell, with *a* ≈ *b* < *c* cell parameters, O_2_ interaction with MAPbI_3_ produces a sizable elongation/contraction of the *a*/*b* cell parameters (*a* = 8.61, *b* = 8.81, *c* = 12.82), respectively. Interestingly, as shown in Table S1 in the ESI,† the formation energy of 4MAPbI_3_·1H_2_O is negative, (–0.45 eV) indicating spontaneous water incorporation into the perovskite lattice. On the other hand, the incorporation of molecular oxygen is unfavourable, with a positive Δ*E*_form_ of 0.17 eV.

The driving force for water incorporation into the perovskite lattice is primarily the formation of hydrogen bonds between the water molecules and lattice iodides, as well as the tight hydrogen bonding interaction between one of the MA cations and the water oxygen atom, as signalled by a ∼1.6 Å separation distance between oxygen and one hydrogen atom of the MA cation (Fig. 3a). Notably, O_2_ interacts only through weaker hydrogen bonds with two vicinal MA cations, reflecting its lower tendency to be intercalated into the perovskite lattice. Water as well as oxygen (although less likely) incorporation into MAPbI_3_ increases the band gap by 50 and 90 meV, respectively, estimated here at the scalar-relativistic level of theory used for geometry optimization (see Table S1† for further details). While this level of theory is only able to accurately calculate the absolute bandgap values of lead halide perovskites due to a fortuitous cancelation of spin–orbit coupling effects,^12^ the calculated variations in bandgap are still valid. Subsequent incorporation of water molecules into MAPbI_3_ was also investigated up to 4 : 4 stoichiometries (see Table S1 and Fig. S6†). The results show that incorporation of a second water molecule has an essentially additive effect on the perovskite properties, with a slight additional volume and bandgap increase as a result of lattice distortion. However, adding four water molecules destabilizes the perovskite lattice, resulting in a large band gap increase. This is consistent with reports of a large band gap hydrated MAPbI_3_ phase with a non-perovskite structure.^21,23,26,29^ These findings support our experimental observations: the crystal edge surface, with a certain degree of distortion with respect to the bulk structure, is prone to water intercalation, resulting in a widening of the bandgap.

## Experimental

### MAPbI_3_ single crystal synthesis

All reagents were purchased from commercial vendors and used as received. Solid PbI_2_ (0.20 g, 0.43 mmol) and methylammonium iodide (0.069 g, 0.43 mmol) were added to 3 mL of acetonitrile. Hydroiodic acid (200 μL, 57 wt%, stabilized with 1.5% hypophosphorous acid) was added to the acetonitrile mixture, which was then sonicated for 5 minutes to ensure complete dissolution of the precursors. Dark, highly reflective block crystals were obtained by allowing diffusion of diethyl ether into this solution over a period of 2 days. The crystals were washed three times with diethyl ether and then dried under reduced pressure. Single crystals were stored under nitrogen and then cleaved along the (110) plane before optical measurements.

### Scanning electron microscopy (SEM)

The sample was placed on conductive carbon tape. SEM observation was carried out using a high vacuum tungsten filament commercial JEOL 6010-LV scanning electron microscope operating at 20 kV.

### Macro-photoluminescence

Time-resolved fluorescence measurements used for registering the spectra and dynamics in Fig. 2 were performed using a femtosecond laser source and a streak camera detection system (Hamamatsu C5680). For details on the experimental set-up see ref. 3 and 19. The measurements were generally carried out under vacuum and the environmental conditions are explicitly stated in the manuscript when other conditions were used.

### Micro-Raman and micro-PL spectroscopy

Our micro-Raman/PL system, used for the measurements presented in Fig. 1b–d and 2f, is based on an optical microscope (Renishaw microscope, see ref. 11 for further details on the experimental apparatus). Samples are excited with a 532 nm laser diode. For the Raman measurements the spectra were registered in the 60–500 cm^–1^ range. The final data were averaged over five hundreds acquisitions in order to improve the signal to noise ratio. The micro-PL was registered simultaneously on the same micrometer-large spot of the sample in back reflection geometry in the range between 700 and 850 nm. To prevent sample degradation or thermal effects, the laser power intensity is kept below 100 μW.

### Computational details

All calculations were carried out with the Quantum Espresso package along with the GGA-PBE functional. Electron–ion interactions were described by scalar relativistic ultrasoft pseudopotentials with electrons from O, N and C 2s, 2p; H 1s; I 5s, 5p; and Pb 6s, 6p, 5d shells explicitly included in the calculations. Variable cell geometry optimizations were carried out including a dispersion correction as in ref. 29. The formation energy of 4MAPbI_3_·*n*H_2_O has been evaluated as follows: Δ*E*_form_ = *E*(4MAPbI_3_·*n*H_2_O) – *E*(4MAPbI_3_) – *n* × *E*(H_2_O), where *E*(4MAPbI_3_·*n*H_2_O) is the energy of the *n*-hydrated perovskite, *E*(4MAPbI_3_) is the energy of the anhydrous perovskite and *E*(H_2_O) is the energy of the isolated water molecule. The same approach has been used to evaluate the formation energy of 4MAPbI_3_·1O_2_.

## Conclusions

In conclusion, we demonstrate that the optical properties and band gap of MAPbI_3_ single crystals are spatially inhomogeneous, particularly at their edge surfaces. The edge surface exhibits a larger band gap and shorter carrier recombination dynamics with respect to the centre. We demonstrate that the origin of this variation is structural in nature, resulting from lattice strain which may be attributed to surface reconstruction at the crystal edges. The degree of strain at the crystal edges can be modulated by exposure to moisture and possibly oxygen to a lesser extent. The sensitivity of the perovskite single crystal optical properties to environmental conditions and the balance between surface and edge effects can explain the discrepancies in optical spectra reported so far in the literature.^5–9^ The crystal edge surface further acts as an open gate for the intercalation of water molecules, occurring spontaneously due to hydrogen bonding interactions with the perovskite lattice. This extends the depth of the surface region further into the bulk of the semiconductor, perhaps resulting in increased carrier trapping in optoelectronic devices. Regarding the moisture-induced degradation of perovskite optoelectronic devices, partial water intercalation into the perovskite structure is likely a necessary first step before structural conversion into a hydrate phase can occur. The observed blue-shift in the PL of perovskite crystals upon humidity exposure may provide an early warning signal before irreversible degradation occurs. Considering the tremendous interest in the application of such materials in optoelectronic devices, where the “interface is the device” (cit. Nobel laureate Herbert Kroemer) this work puts forward important information on the local properties of active interfaces and grain boundaries in polycrystalline perovskite films.
