# Supplementary material for: CH3NH3PbI3 perovskite single crystals: surface photophysics and their interaction with the environment
Source: Chem Sci. 2015 Sep 17;6(12):7305–10. doi: 10.1039/c5sc02542g (PMC5512535; doi:10.1039/c5sc02542g)
Supplement: Supplementary file 1 [file SC-006-C5SC02542G-s001.pdf]

Electronic Supplementary Material (ESI) for Chemical Science.
This journal is © The Royal Society of Chemistry 2015

$CH_3NH_3PbI_3$ Perovskite Single Crystals: Surface Photophysics and its Interaction with the Environment

G. Grancini,[a,] * V. D'Innocenzo,[a, b] E. R. Dohner,[c] N. Martino,[a, b] A. Ram Srimath Kandada, [a] E. Mosconi,[d] F. De Angelis, [d] H. I. Karunadasa,[c] E.T. Hoke,[e] and A.Petrozza[a,] *

†Center for Nano Science and Technology @Polimi, Istituto Italiano di Tecnologia, via Giovanni Pascoli 70/3, 20133, Milan, Italy.

⊥Dipartimento di Fisica, Politecnico di Milano, Piazza L. da Vinci, 32, 20133 Milano, Italy

‡Department of Chemistry, Stanford University, 337 Campus Drive, Stanford, California 94305, USA

◊ Computational Laboratory for Hybrid/Organic Photovoltaics (CLHYO), CNR-ISTM, I-06123, Perugia, Italy

∥ Department of Materials Science and Engineering, Stanford University, 476 Lomita Mall, Stanford, California 94305, USA

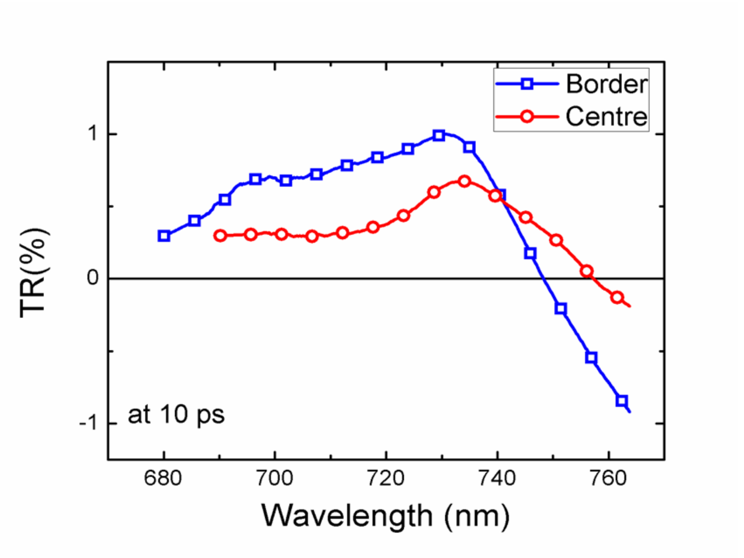

Figure S1. Comparison of the TR spectra at 10 ps time delay moving from the border to the centre of the crystal face under vacuum atmosphere. Focal spot is around 150 μm, excitation wavelength at 640 nm, excitation density around 1μJ/cm^2.

Recently, it has been shown by *Yamada et al* [J. Am. Chem. Soc., 2015, 137 (33), 10456] that red-shifted component of the PL emission in $CH_3NH_3PbI_3$ single crystal results from re-absorption effects induced by fast carrier diffusion within the material. Without ruling out the mentioned effect, we believe that the structural inhomogeneity across the crystal observed by Raman is responsible for the spatial variation of its energetics as revealed by the PL shift in Figure 1d. To corroborate this conclusion we also performed Transient Reflectance (TR) measurements. The TR signal is a sensitive tool for probing the band edge (i.e. monitoring carrier photobleaching at the band edge after carrier thermalization is completed in <1ps) across the single crystal face. Considering the TR spectra at 10 ps time delay (see Figure S1), we observe that moving across the single crystal face (from the border to the centre) the crossing point shifts considerably. This points to an intrinsic inhomogeneity of the band gap across the crystal, as revealed by PL measurements.

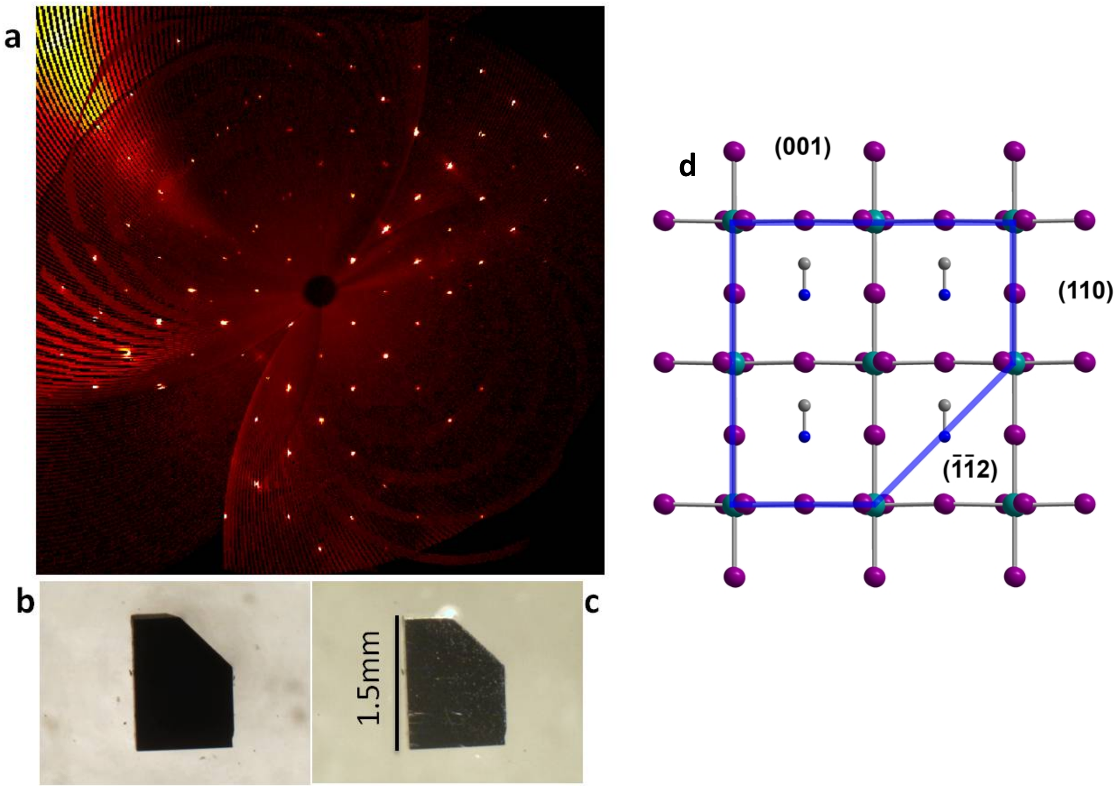

Figure S2 a, Precession X-ray diffraction image of a $MAPbI_3$ single crystal viewed along the [hk0] direction. **b, c,** Micrographs of the $MAPbI_3$ crystal used in the XRD experiment, viewed with episcopic (**b**) and diascopic (**c**) illumination. Schematic (**d**) showing the indexed crystal faces of the $MAPbI_3$ single crystal, viewed with the $(\bar{1}10)$ crystallographic plane in the plane of the page. Note that because of lattice reconstruction typical at crystal edges, the atomic arrangements along each plane in (**d**) do not necessarily reflect the surface termination observed in the actual crystal.

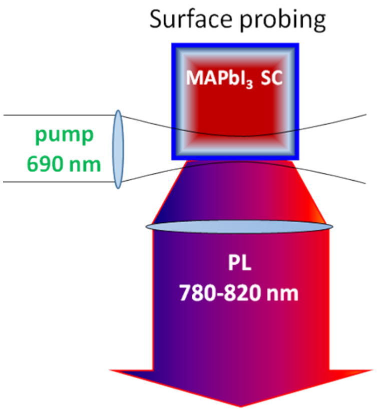

Figure S3 Cartoon of the optical schemes depicting the excitation-collection geometries used for the experiments in Figure 2. In this case we are probing the whole crystal surface PL, averaging the edge contribution.

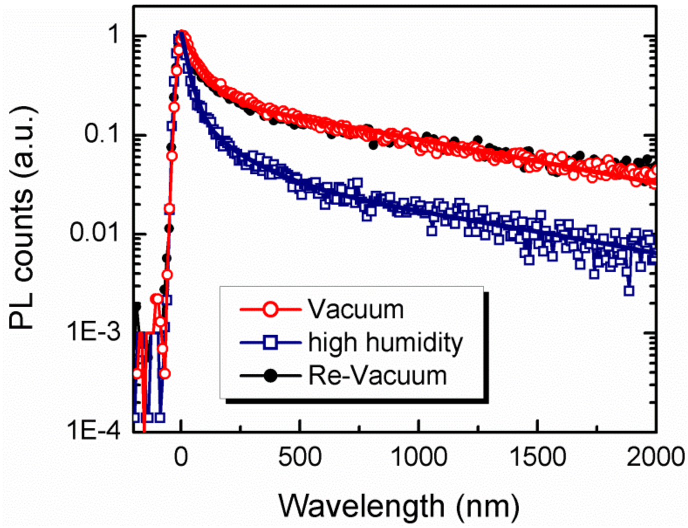

Figure S4 PL emission dynamics of the $MAPbI_3$ crystal measured in vacuum, under exposure to air and after re-evacuation. After the sample chamber is evacuated once more the PL lifetime increases and recovers the original decay dynamics, indicating reversible phenomena.

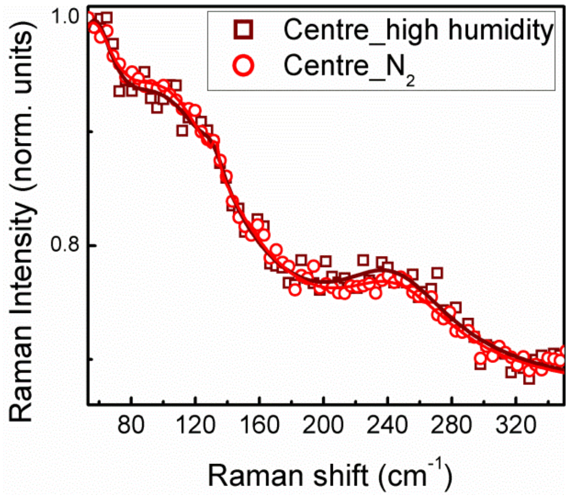

Figure S5 Raman spectra at the center (point A) of the crystal face under different environmental conditions: in dry N_2 or in ambient humid air.

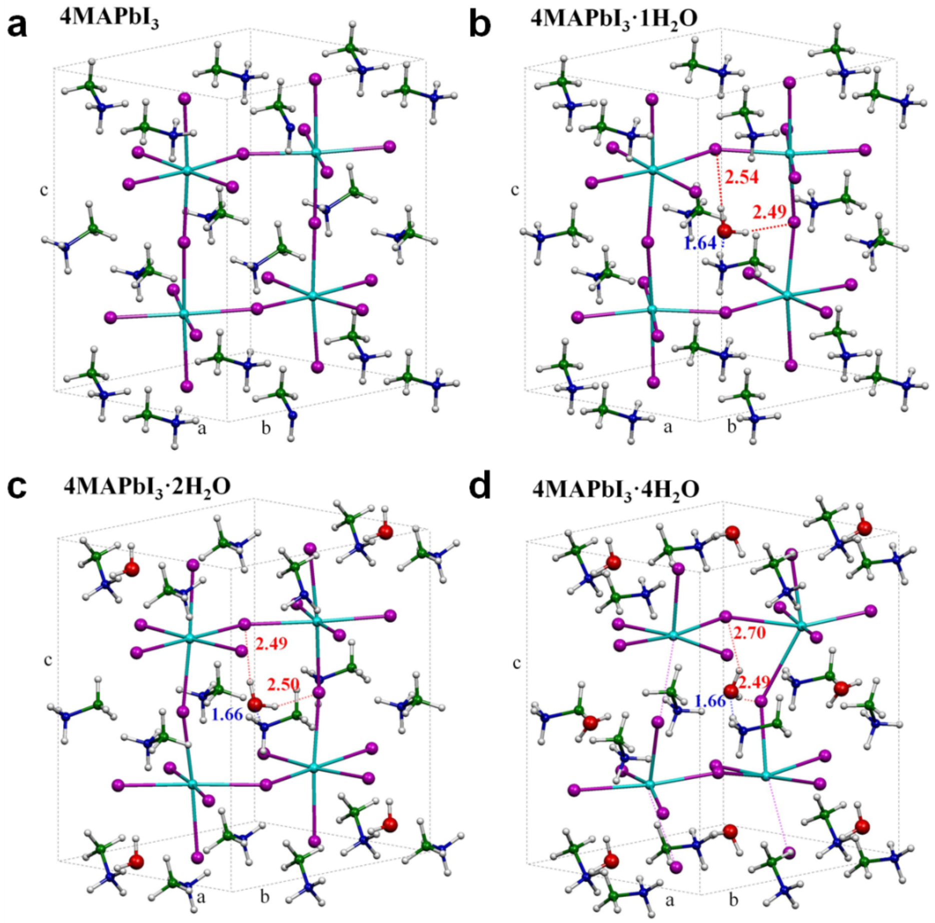

Figure S6 Optimized geometry structures for $4MAPbI_3\cdot nH_2O$ (with n = 0, 1, 2, 4). Pb= light blue, I=violet, green= carbon, blue=nitrogen, red=oxygen, white=hydrogen.
